# Foodborne Pathogens in High-Salt, High-Sugar, and High-Fat Foods: Matrix Effects on Persistence, Adaption and Inactivation for Food Safety

**DOI:** 10.3390/foods15020291

**Published:** 2026-01-13

**Authors:** Yuanmei Xu, Zuhua Liang, Bichao Jia, Zeyi Zuo, Nan Ge, Wenle Yu, Lingtian Wu

**Affiliations:** College of Biological and Food Engineering, Suzhou University of Technology, 99 South Third Ring Road, Changshu 215500, China

**Keywords:** extreme food matrices, low water activity, pathogen persistence, stress adaptation, matrix-dependent inactivation

## Abstract

High-salt, high-sugar, and high-fat foods are popular among consumers because of their distinctive sensory qualities and extended shelf stability. Although these matrices have long been regarded as inhospitable to microbial proliferation, numerous outbreaks linked to salted meats and fish, chocolate, tahini, peanut butter, and cheese demonstrate that such environments can nevertheless support prolonged pathogen survival and complicate inactivation efforts. This review compiles reported outbreaks and recalls associated with these products and shows that *Salmonella* spp., *Listeria monocytogenes* (*L. monocytogenes*), *Escherichia coli* (*E. coli*), *Staphylococcus aureus* (*S. aureus*), and *Vibrio parahaemolyticus* (*V. parahaemolyticus*) are the principal pathogens involved. It further examines key factors shaping survival and the mechanisms underlying pathogen persistence in these extreme matrices. Growing evidence also indicates that elevated levels of salt, sugar, and fat can modulate the effectiveness of inactivation technologies: salt may exert both inhibitory and sensitizing effects, whereas sugar and fat generally provide protective advantages during treatment. Clarifying these matrix-dependent interactions is critical for designing optimized multi-hurdle preservation approaches that ensure microbial safety while maintaining product quality in extreme foods.

## 1. Introduction

Foodborne pathogens remain a major global threat to public health and food safety, causing millions of illnesses, hundreds of thousands of deaths, and substantial economic losses worldwide each year [1]. Despite advances in food processing and preservation technologies, pathogen contamination still continues to occur in a wide range of products. Foods with high levels of salt, sugar, or fat, such as salted fish and cured meat, chocolate, peanut butter, and cheese, have been repeatedly associated with microbial contamination and foodborne outbreaks [2]. While these foods are favored by consumers for their distinctive flavors, appealing textures, and extended shelf life, their unique physicochemical properties create special microenvironments that influence microbial survival and inactivation.

Traditionally, the extreme conditions characteristic of these foods, including low water activity (*a_w_*), high osmotic pressure and limited nutrient diffusion, have been considered unfavorable for microbial growth [3,4,5]. However, increasing evidence from experimental studies and outbreak investigations indicates that certain foodborne pathogens can persist, and even adapt, within these extreme matrices [6]. For example, low *a_w_* induced by high levels of salt or sugar can increase microbial resistance to thermal inactivation, while lipid-rich matrices may form a protective layer that shields cells from physical or chemical treatments [7,8]. Conversely, elevated salt or fat levels can sensitize cells to inactivation approaches under certain conditions, highlighting the complex and context-dependent nature of these interactions [9,10].

Given the paradoxical effects of salt, sugar, and fat on both suppressing and protecting foodborne pathogens, a systematic understanding of microbial survival, adaptation mechanisms and inactivation in these extreme food matrices is essential. Such knowledge underpins effective intervention strategies, optimization of multi-hurdle technologies, and improved risk assessment in the food industry. In contrast to existing reviews that predominantly focus on a single stress factor (e.g., low *a_w_*) or a specific food category [2,11], this review provides an integrated, matrix-oriented perspective on pathogen persistence and inactivation in high-salt, high-sugar, and high-fat foods. By systematically comparing these extreme matrices, this review highlights both shared and distinct survival mechanisms, clarifies how matrix composition modulates the efficacy of thermal and non-thermal inactivation strategies, and identifies key knowledge gaps to guide future research and the development of matrix-tailored control approaches.

In preparing this review, we focused on studies that provide mechanistic insights, outbreak evidence, and implications for microbial inactivation in high-salt, high-sugar, and high-fat foods. Relevant publications were identified through targeted searches of major databases (e.g., Web of Science and Google Scholar) using combinations of keywords related to foodborne pathogens, extreme food matrices, stress adaptation mechanisms and inactivation technologies. Priority was given to recent and highly cited studies, as well as research that offered clear mechanistic or technological perspectives. Significant earlier works were also retained when they contributed fundamental concepts that could not be replaced by newer studies.

## 2. Bacterial Contamination and Survival Characteristics in Extreme Food Matrices

The term “extreme food matrices” refers to food matrices whose physicochemical conditions differ markedly from conventional growth media, thereby imposing substantial stress on microbial cells. In this review, the term specifically refers to high-salt, high-sugar, and high-fat foods. Owing to these distinct physicochemical characteristics, extreme food matrices can differentially influence the persistence of foodborne pathogens. The following section therefore summarizes reported bacterial contamination and survival behavior associated with high-salt, high-sugar, and high-fat foods.

### 2.1. Bacterial Contamination and Survival Characteristics in High-Salt Foods

#### 2.1.1. Bacterial Contamination of High-Salt Foods

High-salt foods encompass a broad range of moisture conditions and can be broadly categorized into high-salt, high-moisture and high-salt, low-moisture products. High-salt, high-moisture foods, such as pickled vegetables, no-dried salted meats or fish, and miso, retain substantial amounts of water, with salt predominantly present in dissolved form, also resulting in relatively high *a_w_*, typically above 0.85 [12]. Notably, soy sauce represents an exception, as its *a_w_* is often below 0.85 despite its high moisture content, owing to the high concentration of dissolved solutes that strongly bind available water [13]. In these systems, microbial inhibition is primarily driven by elevated osmotic pressure and ionic imbalance. In contrast, high-salt, low-moisture foods, including salted dried fish, dried cured meats, dried seafood, and salt-roasted nuts, are produced through the combined application of salting and dehydration. These products are characterized by markedly reduced *a_w_* (often <0.85), high osmotic pressure, ionic imbalance and restricted mass transfer, which together impose more severe constraints on microbial survival and growth [6,14,15]. Although high salt levels were effective at inhibiting microbial proliferation, outbreaks associated with salted products have demonstrated that salting alone cannot ensure food safety. Several foodborne pathogens are able to persist under high-salt conditions, posing significant risks (Table 1).

*L. monocytogenes* exhibits notable tolerance to low temperatures and moderate salinity (up to 10% NaCl), allowing it to survive in cold-smoked fish. It has repeatedly been implicated in outbreaks involving salmon and trout across Europe and the UK [29]. *S. aureus* can grow in environments containing up to 15% NaCl and produce enterotoxins under favorable conditions. In 2019, the Centre for Food Safety of Hong Kong also reported high levels (~150,000 CFU/g) of *S. aureus* in bottled salted egg paste, leading to a large-scale recall [6,19]. Although less halotolerant than *L. monocytogenes* or *S. aureus*, *Salmonella* has been isolated from cold-smoked salmon and salt-cured meats, such as salami and antipasto, particularly in cases of inadequate salting or post-processing contamination. In one study, *Salmonella* survived for up to 60 days in 80% salted horse mackerel fillets [30]. Similarly, Shiga toxin-producing *E. coli* (STEC) has also been linked to moderately salted fermented vegetables, such as kimchi, with a recent outbreak in Canada (2022) caused by STEC O157:H7 from salted Napa cabbage kimchi, resulting in multiple confirmed cases [25]. *V. parahaemolyticus*, a halotolerant species, has also been associated with salted or fermented seafood, notably soy sauce–marinated crabs in Korea [28]. Collectively, these findings highlight that salting alone cannot guarantee food safety. Effective control requires complementary preservation strategies together with stringent hygiene and monitoring practices throughout production and distribution.

#### 2.1.2. Microbial Survival in High-Salt Foods

Salt generally inhibits the growth of many microorganisms. For example, Busnello et al. reported that an 8 log CFU/g inoculum of *Salmonella* in pork feet containing 25% salt became undetectable after 26 days at 10 °C [31]. However, contamination cases indicate that various pathogens can still persist in high-salt foods [16]. Studies have illustrated that a combination of physicochemical factors influences pathogens survival in high-salt environments, including salt concentration, *a_w_*, microbial species, storage temperature, and prior environmental adaptation.

While increasing salt concentrations generally exert stronger inhibitory effects on microbial growth, Shrestha et al. found that the survival of *Salmonella* in low-*a_w_* chicken paste and powder stored at 21 °C for extended periods was not significantly affected by elevated salt levels (33%) [32]. This may be because *a_w_* is the primary factor governing microbial survival, and the increase in salt concentration only slightly affects the *a_w_* of chicken powder. Microbial species exhibit different levels of salt tolerance. Cho et al. reported that *S. aureus* survived for 28 days at both 5 and 22 °C in seafood containing high-salinity soy sauce, showing greater salt tolerance than Gram-negative bacteria. They also observed that foodborne pathogens survived longer in high salt food matrix at lower storage temperature (5 °C) [33].

Pre-adaptation or cross-protection also plays a significant role. Pittman et al. demonstrated that *L. monocytogenes* exposed to cold temperatures prior to salt treatment exhibited enhanced survival in NaCl [34]. Similarly, acid-adapted *V. parahaemolyticus* [35], *E. coli* [36] and *S.* Typhimurium [37] showed significantly higher salt tolerance than non-adapted cells, while desiccated *S*. Typhimurium displayed greater salt resistance than non-desiccated counterparts [38], suggesting environmental stresses encountered during food processing can induce cross-protection, enhancing microbial survival under salt stress.

Collectively, these studies indicate that microbial survival in high-salt foods is influenced by multiple interrelated factors. Effective control therefore requires consideration of salt concentration, microbial species, storage conditions, and potential pre-adaptation, often necessitating combined preservation strategies to ensure food safety.

### 2.2. Bacterial Contamination and Survival Characteristics in High-Sugar Foods

#### 2.2.1. Bacterial Contamination of High-Sugar Foods

Sugars in high sugar products may be naturally present or added during processing. According to *a_w_*, high sugar foods can be divided into high-sugar, high-moisture and high-sugar, low-moisture foods. High-sugar, high-moisture foods with relatively high *a_w_* (often >0.85), include sugar-sweetened beverages (e.g., fruit juices, fresh fruit products, energy drinks) and fruit fillings, sweetened dairy products (e.g., sweetened yogurt, and ice cream), and moist bakery items (e.g., moist pastries, sweet rolls, and muffins). Although the osmotic pressure imposed by dissolved sugars partially inhibits microbial growth, the high free water availability still allows many bacteria to survive or grow [39,40]. In contrast, high-sugar, low-moisture products, characterized by markedly reduced *a_w_* (often <0.85), include dried fruits, jams, candies, chocolate, tahini halva, cookies, and honey. In these matrices, the proliferation of most microorganisms is restricted by high osmotic and desiccation stresses, while in some cases, high viscosity (e.g., in honey) further limits microbial movement and nutrient diffusion [41,42,43]. Despite the strict conditions produced by high sugar, osmotolerant microorganisms are capable of surviving under such conditions by entering stress-adapted physiological states, thereby enabling their persistence during extended storage [44] (Table 2).

*Salmonella* can persist in chocolate for prolonged periods and even withstand thermal processing, posing significant safety risks, as demonstrated by the *S.* Typhimurium outbreak linked to Kinder products across multiple countries in 2022 [45,46]. Similarly, an outbreak of *S.* Agbeni infections in Norway during 2018–2019, linked to dried fruits, resulted in 56 confirmed cases [47]. Moreover, high-sugar confections like tahini halva have also been subject to recalls due to *Salmonella* contamination, often originating from raw sesame seeds or post-processing contamination [48]. In addition to *Salmonella*, *S. aureus* has also been isolated from sucrose-rich products such as raisins, tahini halva, and handmade sweets [49].

In contrast, high-moisture, high-sugar foods, such as sweetened beverages, fresh fruits, and ice cream provide favorable conditions for pathogen growth. *L. monocytogenes* has been detected in ice cream and frozen dessert products, leading to illnesses and a nationwide recall [50]. Furthermore, 13 confirmed cases of *E. coli* O111 infection in California were linked to unpasteurized apple juice in 2015 [51]. Collectively, these cases highlight that both low-moisture and high-moisture high-sugar foods pose microbiological risks. Thus, increasing sugar content alone cannot ensure microbiological safety, underscoring the need for integrated inactivation treatments and strict processing hygiene.

**Table 2:** Cases of microbial contamination in high-sugar foods.

Foods	Strains	Country/Year	Case/Deaths	References
Chocolate products	*S.* Typhimurium	EU/EEA countries, UK/2022	150/0	[52]
Dried fruits	*S.* Agbeni	Norway/2018–2019	56/0	[47]
Tahini halva	*Salmonella* spp.	Canada/2021	Recalls	[48]
Honey smacks cereal	*S.* Mbandaka	USA/2018	135/0	[53]
Ice cream	*L. monocytogenes*	US/2023	2/0	[50]
Apple Juice	*E. coli* O111	USA/2015	13/0	[51]

#### 2.2.2. Microbial Survival in High-Sugar Foods

The contamination cases above clearly indicate that microorganisms can survive for prolonged periods in high-sugar matrices. Among them, *Salmonella* spp. are the most frequently reported pathogens associated with high-sugar foods, particularly low-moisture products such as chocolate, dried fruits, jams, and tahini halva. Their remarkable desiccation resistance enables survival for months to years in low *a_w_* environments [54]. The survival of pathogens in high-sugar foods is influenced by several factors, including the sugar type, *a_w_*, and storage conditions. For example, Maniscalco et al. demonstrated that different sugar types exert distinct inhibitory effects on bacteria such as *S. aureus*, *E. coli*, and *Salmonella* enterica, even at the same concentrations [55]. Storage temperature also plays a critical role in long-term persistence. Beuchat et al. reported that *Salmonella* survived in sucrose for at least 52 weeks, showing higher viability at 5 °C than at 25 °C [49]. Similarly, *Salmonella* persisted longer in aqueous homogenates of dried fruits at 4 °C than 5 °C [56]. In addition, *L. monocytogenes* has been shown to survive on raisins and dried strawberries for up to 336 days at 4 °C [57]. These findings suggest that refrigeration, rather than reducing microbial risk, may actually prolong pathogen survival in high-sugar foods. Furthermore, Beuchat et al. also found the tolerance of *Salmonella* to high sugar and low *a_w_* increased when habituation to low *a_w_* preceded exposure to osmotic shock, indicating that pre-exposure to a dry environment induces cross-resistance [49]. Overall, microbial survival in high-sugar foods is influenced by a combination of factors; therefore, effective risk control requires consideration of these interacting factors rather than reliance on reduced *a_w_* alone.

### 2.3. Bacterial Contamination and Survival Characteristics in High-Fat Foods

#### 2.3.1. Bacterial Contamination of High-Fat Foods

High-fat foods are generally defined as food products in which fat accounts for more than 20–30% of the total caloric content or weight. Based on *a_w_*, these foods can be classified into two groups. The first group, high-moisture, high-fat foods, includes processed meats, cheeses, creams, shakes, diluted tahini and high-fat dairy spreads, typically providing favorable conditions for the growth of pathogenic microorganisms, such as *L. monocytogenes* and *S. aureus*. In contrast, low-moisture, high-fat foods, such as dried nuts, chocolate, peanut butter, tahini, and fat-based powders, generally have *a_w_* values below 0.70. In some systems (e.g., tahini and peanut butter), the hydrophobic and high viscous nature of the lipid-continuous matrix physically restricts microbial mobility as well as access to free water and nutrients [58,59]. Therefore, microbial growth is limited in these matrices. However, the high lipid content presents unique challenges for food preservation and pathogen inactivation, as lipid-rich matrices can shield microbial cells from environmental stress. Consequently, high-fat foods have frequently been associated with microbial contamination and foodborne outbreaks across various product categories [2,46,60] (Table 3).

In particular, multiple outbreaks and product recalls in recent years highlight *Salmonella* as the primary microbiological hazard in high-fat, low-moisture foods, including peanut butter, tahini, and chocolate products [61]. Moreover, recent recalls have highlighted that the presence of *L. monocytogenes*, which is less common than *Salmonella*, continues to pose a safety risk in high-fat products, including peanut butter, nut spreads, cheese, and supplemental shakes [62,63]. *S. aureus* has also occasionally been detected in cheese and cream-based desserts, where its enterotoxins can remain active even after bacteria are inactivated, posing significant health risks to consumers [64,65]. Overall, the contamination of high-fat foods with pathogenic microorganisms remains an urgent public health concern, underscoring the need for enhanced preventive measures and greater public awareness.

**Table 3:** Cases of microbial contamination in high-fat foods.

Foods	Strains	Country/Year	Case/Deaths	Reference
Pistachio cream	*Salmonella*	USA/2025	4/0	[66]
Peanut butter and peanut products	*Salmonella*	USA/2006/2008/2012/2022	1484/9	[67]
Chocolate products	*S.* Typhimurium	EU/EEA countries and the UK/2022	150/0	[52]
Tahini or halva	*S.* Amsterdam, *S.* Havana, *S.* Kintambo, *S.* Mbandaka, *S.* Orion, and *S.* Senftenberg	Germany, Sweden, Norway, Netherlands, Canada, USA, New Zealand/ 2019–2022	121/0	[68]
Hummus	*Salmonella*	Canada/2020	45/0	[69]
Tahini (sesame paste)	*S.* Concord	USA/2018	8/0	[70]
Dried coconut	*S.* Typhimurium	USA/2018	14/0	[71]
Traditional hand-crafted cheese	*S. aureus*	Switzerland/2018	3/0	[64]
Chantilly cream dessert	*S. aureus*	Italy/2015	24/0	[65]
Fresh, soft Hispanic-style cheese	*L. monocytogenes*	USA/2021	13/1	[62]
Supplement shakes	*L. monocytogenes*	USA/2018–2025	42/14	[72]

#### 2.3.2. Microbial Survival in High-Fat Foods

The survival strategies of foodborne pathogens differ markedly between high-moisture and low-moisture, high-fat foods. In high-moisture, high-fat products, the relatively high *a_w_* enables active growth of pathogens such as *L. monocytogenes*, *S. aureus*, and *Salmonella* spp. When temperature control or hygiene measures are inadequate, these foods present a particularly high risk because they not only protect microbial cells but also provide sufficient moisture to support bacterial proliferation [73]. In contrast, high-fat, low-moisture foods impose unique stresses on microbial cells due to the combined effects of low *a_w_* and limited nutrient diffusion. Despite these harsh conditions, several foodborne pathogens, including *S. enterica*, *L. monocytogenes*, and *S. aureus*, can persist for prolonged periods in such matrices. For instance, a cocktail of *Salmonella* serotypes (*S.* Cubana, *S.* Aberdeen, *S.* Typhimurium, and *S.* Paratyphi) survived in tahini samples with *a_w_* values of 0.17, 0.35, and 0.50 for up to 12 months at 10 °C and 9 months at 25 °C, respectively. These findings indicate that lower storage temperatures favor the long-term survival of foodborne pathogens in high-fat, low-moisture foods [74].

In addition to storage temperatures, *a_w_* also plays a particularly crucial role in pathogen survival in high-fat, low-moisture foods. Kataoka et al. reported that *Salmonella* survived better in peanut paste at an *a_w_* of 0.3 than at 0.6 [75]. In addition, the relative proportions of fat and carbohydrate influence pathogen survival. He et al. demonstrated that *Salmonella* persisted longer in peanut butter formulations with lower fat and higher carbohydrate contents [76]. Consistently, peanut butter containing 33.33% fat and 41.67% carbohydrate supported greater *S. enterica* survival than formulations with 50% fat and 21.9% carbohydrate during a four-week storage period, suggesting that carbohydrates exert a protective effect for foodborne pathogens in these foods [77].

Collectively, these findings underscore the multifactorial nature of pathogen survival in high-fat, low-moisture foods and highlight the complex interplay among storage temperature, *a_w_*, and nutrient composition that governs microbial persistence.

## 3. Survival Adaptive Mechanisms in Extreme Foods

**Figure 1:**
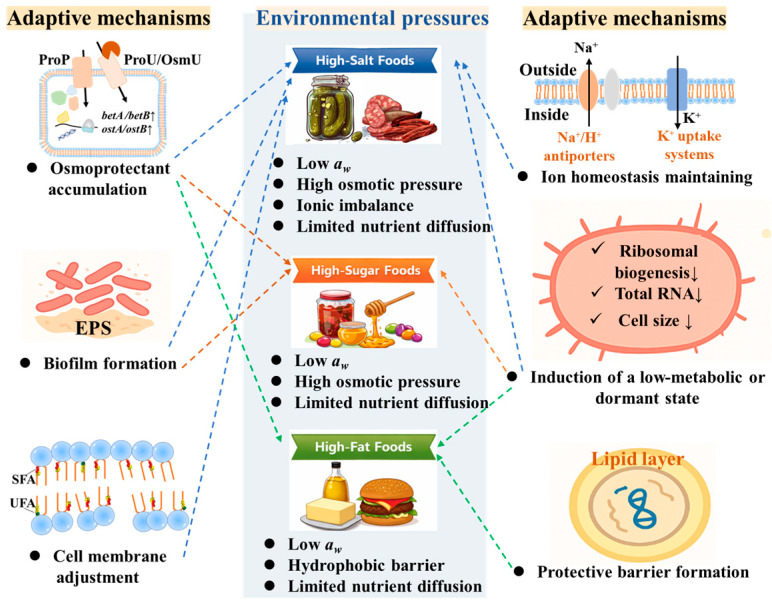
Environmental pressures and survival mechanisms of food pathogens in extreme foods [3,6,78,79,80,81,82,83,84,85,86,87,88,89,90,91,92,93,94,95,96,97,98].

High-salt, high-sugar, and high-fat foods impose distinct environmental pressures that critically shape the survival of foodborne pathogens. In response, microorganisms employ a combination of shared and stress-specific adaptive mechanisms to cope with reduced *a_w_*, osmotic stress, ionic imbalance, and physical constraints imposed by different food matrices. While certain strategies, such as osmoadaptation, stress-responsive gene regulation, and entry into low-metabolic or dormant states, are conserved across multiple extreme environments, other responses are tailored to specific stresses, including ionic toxicity in high-salt foods or hydrophobic isolation in high-fat matrices. The following sections systematically describe these common and distinct adaptive mechanisms that enable pathogens to persist in high-salt, high-sugar, and high-fat foods (Figure 1).

### 3.1. Survival Adaptive Mechanisms in High-Salt Foods

#### 3.1.1. Osmoprotectants Accumulation

While survival behaviors of foodborne pathogens in high-salt foods are well documented, their adaptive mechanisms are crucial to understanding halotolerance. The primary strategy is the intracellular accumulation of osmoprotectants, such as glycine betaine, proline, glutamate, and trehalose [78]. These molecules do not interfere with cellular metabolism but help maintain a counterbalance external osmotic pressure and preserve enzyme function under osmotic stress. For example, *Salmonella* exhibited significantly higher intracellular levels of trehalose, proline, and betaine to adapt to high-salt environments, accompanied by the upregulation of the related key genes including *proU*, *proV*, *kdpC*, *kuP*, and *rpoS* [3,79]. In *S. aureus*, the level of L-proline increased under 10% NaCl environments, and genes such as *betA* and *betB*, involved in the betaine biosynthesis pathway, were activated to promote betaine synthesis and transport [6,80]. Similarly, a recent study showed that the transcriptional factor MntR mediates hyperosmotic stress resistance of *L*. *monocytogenes* in high-salt condition by positively regulating the glycine betaine uptake system Gbu [81]. *V. parahaemolyticus* survives in high-salt environments by employing multiple compatible solute uptake systems, including ABC-family transporters (ProU1 and ProU2) and BCCT-family transporters, as well as the biosynthesis of betaine and ectoine, thereby maintaining osmotic balance in marinated crabs [82].

Overall, these findings indicate that the ability of foodborne pathogens to accumulate or synthesize compatible solutes, coupled with the activation of multiple osmoprotective transport and metabolic pathways, represents a conserved and critical strategy for survival under high-salt stress.

#### 3.1.2. Ion Homeostasis Maintenance

In response to stress imposed by high-salt food environments, foodborne pathogens employ a combination of Na^+^/H^+^ antiporters and K^+^ uptake systems to maintain intracellular ionic balance, membrane potential, and proton motive force (PMF), all of which are essential for survival. In halotolerant bacteria, finely regulated Na^+^/H^+^ antiporters such as NhaA, NhaB, NhaC, MnhF, and NhaD expel excess cytoplasmic Na^+^ in exchange for protons, thereby preventing sodium toxicity and cytoplasmic alkalinization. For example, *V. parahaemolyticus* mutants lacking *nhaA* or *nhaD* exhibit markedly impaired growth under high-salt conditions [83]. Similarly, in *Vibrio cholerae*, genes encoding the symporters gltP and gltS, responsible for co-transporting one glutamate molecule along with H^+^/Na^+^, were significantly downregulated under salt stress, whereas the trkH system responsible for K^+^ uptake was consistently upregulated in salt-tolerant strains [84]. In *L. monocytogenes*, osmotic upshift triggers a rapid accumulation of intracellular K^+^, followed by compatible solute uptake to restore turgor pressure [85]. Likewise, *S. aureus* utilizes multiple systems to maintain Na^+^/K^+^ homeostasis: under elevated NaCl, the Ktr (K^+^ transporter system) and Kdp (K^+^-dependent ATPase system), together with Mnh1/Mnh2 Na^+^/H^+^ antiporters, act in coordination to stabilize intracellular ion levels and sustain PMF [86]. Overall, these findings highlight that foodborne pathogens rely on a synergistic network of Na^+^/H^+^ antiporters and K^+^ transport systems as a frontline strategy to maintain ion homeostasis and ensure survival under high-salt stress.

#### 3.1.3. Cell Membrane Adjustment

Bacterial cell membranes can adapt to extreme environments by modulating their lipid composition to sustain normal growth and physiological functions [87]. Under osmotic stress, many salt-tolerant pathogens remodel their membrane lipid profiles to preserve stability and functionality. For instance, *L. monocytogenes* increases the ratio of diphosphatidylglycerol (DPG) to phatidylglycerol (PG), which stabilizes membrane bilayer structures and prevents the formation of disruptive non-bilayer phases [88]. Similarly, the fatty acid composition of *S. aureus* and *E. coli* shifts under high-salt conditions, with an increased ratio of unsaturated to saturated fatty acids (UFA/SFA) observed at 3% NaCl compared to 0% NaCl [89]. Furthermore, salt-tolerant *Salmonella* strains can maintain elevated membrane potential in hyperosmotic environments, facilitating ionic channel activity and transport system expression, thereby preserving cellular integrity and viability [79]. Collectively, these observations suggest that halotolerant bacteria enhance their survival in high-salt environments through dynamic remodeling of membrane lipids and fatty acids, which maintains membrane stability, fluidity, and essential physiological functions. Nevertheless, current understanding of the molecular mechanisms governing membrane adaptation to high-salt stress remains limited, warranting further investigation.

#### 3.1.4. Biofilm Formation

In high-salt food matrices, the ability of foodborne pathogens to form biofilms represents a crucial survival strategy that complements their cellular osmotic adaptation mechanisms. Under hyperosmotic conditions caused by high salinity (e.g., 10% NaCl), *Salmonella* strains retain the capacity to form biofilms, which stabilize their local microenvironment by retaining moisture, maintaining membrane integrity, and accumulating osmoprotectants [91]. Similarly, under salt stress, *S. aureus* enhances biofilm development through increased production of extracellular polysaccharides (EPS). Transcriptome analyses have revealed that genes such as cap5A, cap5B, and cap8C, which are involved in the biosynthesis of capsular polysaccharide (CPS), which is the primary protective component of EPS, are upregulated in response to high salinity, directly promoting biofilm formation [92]. Furthermore, proteomic studies have shown that *S. aureus* exhibits elevated levels of IsaA, a key protein associated with biofilm stability, under high NaCl conditions [93]. Overall, biofilm formation provides an additional layer of protection that enables pathogens to withstand osmotic stress, thereby enhancing their persistence in salted foods and increasing potential risks to food safety.

#### 3.1.5. Induction of the VBNC State

Exposure to high-salt environments can induce foodborne pathogens to enter the viable but non-culturable (VBNC) state, which is generally regarded as a reversible, low-metabolic and dormancy-like survival strategy rather than a true dormant state. In the VBNC state, cells exhibit reduced size while maintaining metabolic activity, intact membranes, and the genetic potential for resuscitation under favorable conditions, yet they lose the ability to grow on conventional culture media [94]. The emergence of VBNC pathogens in salted foods poses a significant threat to public health and food safety. For instance, *V. parahaemolyticus* can transition into the VBNC state under extreme salt stress when exposed to 30% NaCl condition combined with low temperature (4 °C), accompanied by an increased proportion of saturated fatty acids, reduced membrane potential, and morphological transformation into coccoid forms [95]. Similarly, *E. coli* O157:H7 can enter the VBNC state under high salinity conditions during salted fish processing, while still expressing pathogenic genes (*stx1*, *stx2*, *eae*, *hly*), thereby maintaining its pathogenic potential [96]. In addition, *Salmonella* has been reported to enter the VBNC state when exposed to 7% Nl, with decreased intracellular RpoS levels observed during VBNC induction [97,98].

In summary, foodborne pathogens adapt to high-salt environments through multiple survival strategies, including the accumulation of osmoprotectants, maintenance of ion homeostasis, membrane lipid remodeling, biofilm formation, and transition to the VBNC state. These mechanisms not only enable pathogens to endure osmotic stress but also help preserve their virulence, posing hidden and persistent risks to food safety.

### 3.2. Survival Adaptive Mechanisms in High-Sugar Foods

Despite increasing concern over foodborne pathogen contamination in high-sugar foods, mechanistic studies on microbial adaptation in such environments remain limited. In contrast, pathogen survival under high-salt conditions has been extensively investigated, as reviewed in Section 3.1. Both high-sugar and high-salt environments impose osmotic stress resulting from reduced *a_w_*, and it is generally recognized that foodborne pathogens employ similar adaptive strategies under these conditions, including the accumulation of compatible solutes, biofilm formation, and transition into the VBNC state. For instance, genes related to trehalose synthesis, such as *otsA* and *otsB* in *Salmonella enterica* were significantly upregulated in chocolate powder during 12 weeks of storage [99]. Zheng et al. demonstrated that *Salmonella* Typhimurium adapts to high-glucose conditions by modulating its glucose transport and metabolic pathways and enhances biofilm formation through the upregulation of genes associated with the two-component regulatory system [100]. Likewise, Jayeola et al. found that *Salmonella* in dried apples or apple juice supplemented with 30% fructose entered the VBNC state after five days of storage at 25 °C, whereas no VBNC cells were detected in unsupplemented apple juice [101]. Collectively, these results highlight the need for further mechanistic investigations into pathogen adaptation to high-sugar stress to better inform microbial risk assessment and control strategies.

### 3.3. Survival Adaptive Mechanisms in High-Fat Foods

Despite increasing awareness of foodborne outbreaks associated with high-fat, low-moisture foods, mechanistic studies on pathogen survival in these matrices remain limited. It is well established that lipid matrices can form a protective barrier, mitigating oxidative damage, desiccation stress, and direct exposure to environmental stresses, thereby promoting the survival of foodborne pathogens in high-fat, low-moisture foods [102]. However, few studies have systematically examined how exposure to the combined stressors of low *a_w_* and high fat affects bacterial morphology, physiology, and gene expression.

These general protective mechanisms of lipid matrices have been supported by limited experimental observations, revealing how pathogens adapt at the cellular and molecular levels when exposed to high-fat environments. Zhao et al. reported that *Salmonella enterica* Enteritidis PT 30 maintained its characteristic rod-shaped morphology during prolonged storage in peanut oil but displayed dense aggregation and irregular clustering [103]. The cells remained motile within intact vacuoles, indicating residual metabolic activity. This report further showed that exposure to the oil environment also caused a marked suppression of ribosomal biogenesis and oxidative phosphorylation, driving the cells into a metabolically dormant state. Furthermore, KEGG pathway enrichment analysis revealed significant upregulation of genes related to flagellar assembly and bacterial chemotaxis, suggesting the presence of active motility mechanisms despite metabolic dormancy. Similarly, Deng et al. reported that *Salmonella* cells in peanut oil entered a physiologically dormant state, with less than 5% of the genome being transcribed, accompanied by accelerated RNA degradation as a strategy to acquire nutrients and sustain survival in the oil matrix [104]. Fong and Wang also observed that incubation in peanut oil upregulated genes related to trehalose synthesis (*otsB*) and catabolism of long-chain fatty acids (*fadA*), thereby enhancing the survival of strains in these foods [105]. Overall, foodborne pathogens adapt to lipid-rich and low *a_w_* environments by entering a metabolically dormant state, suppressing energy-intensive processes while selectively maintaining stress resistance and motility through targeted gene regulation. In addition, the accumulation of compatible solutes, such as trehalose, further enhances microbial adaptation to high-fat environments. Nevertheless, the precise regulatory networks remain to be fully elucidated.

## 4. Impact of High-Salt, High-Sugar, and High-Fat Matrices on Inactivation Efficiency

### 4.1. Impact of High-Salt Matrices on Inactivation Efficiency

**Figure 2:**
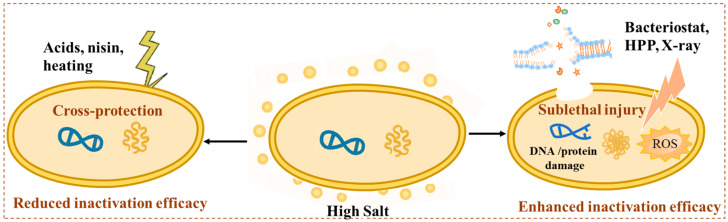
Impact of high-salt matrices on inactivation efficiency [9,106,107,108,109,110,111,112,113,114].

High-salt environments exert complex effects on the efficacy of foodborne pathogen inactivation technologies (Figure 2). On the one hand, elevated salt levels can enhance the effectiveness of certain treatments by inducing sublethal injury, increasing cell permeability, and disrupting membrane integrity, thereby rendering pathogens more susceptible to organic acids, oxidants, and hurdle-based approaches. For instance, NaCl was reported to enhance the bactericidal actions of carvacrol, thymol, and acetic acid against halotolerant species such as *E. coli* O157:H7, *L. monocytogenes*, and *S. aureus* [106,107]. Likewise, Huang et al. demonstrated that combining high-pressure processing (HPP) with 3% NaCl immersion significantly enhanced microbial inactivation and concurrently improved texture stability, reducing lipid oxidation, and preserving color and sensory quality [9]. Seok and Ha further observed that pretreatment with 15% NaCl increased the susceptibility of *L. monocytogenes* and *S. aureus* to X-ray irradiation by elevating reactive oxygen species (ROS) generation and interrupting peptidoglycan synthesis, thereby improving bactericidal efficiency [108]. Additionally, *Bacillus cereus* endospores formed under 7% NaCl exhibited reduced heat resistance due to decreased hydrophobicity, membrane fluidity, and spore density [109].

Conversely, salt-induced reduction of *a_w_* can protect pathogens by enhancing their thermal resistance and diminishing the efficacy of heat-based treatments [110]. Moreover, prior exposure to elevated salt levels may confer cross-protection against other stresses. For example, *L. monocytogenes* in meat and fish products showed increased resistance to nisin and heat following high-salt adaptation [111,112]. Similarly, *L. monocytogenes* pre-exposed to BHI supplemented with 6% NaCl at 7 °C showed enhanced nisin resistance through LiaR-dependent transcriptional regulation [113]. Furthermore, 3% salt has also increased the resistance of *E. coli* O157:H7 to acetic, propionic, and lactic acid; however, when combined with malic, tartaric, citric, or phosphoric acid, it enhanced the reduction of *E. coli* O157:H7 and *S.* Typhimurium compared with acid treatment alone, indicating that synergistic inactivation depends on the acid type [114]. Overall, the dual role of salt in microbial inactivation, as both an enhancer and a protector, underscores the need to optimize salting conditions and integrate multiple preservation strategies to ensure microbial safety in high-salt foods.

### 4.2. Impact of High-Sugar Matrices on Inactivation Efficiency

**Figure 3:**
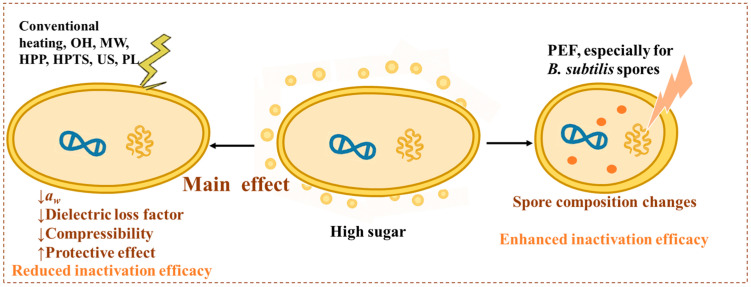
Impact of high-sugar matrices on inactivation efficiency [115,116,117,118,119,120,121,122,123,124,125].

High-sugar environments exert a pronounced impact on the efficacy of microbial inactivation treatments (Figure 3). Elevated sugar concentrations decrease *a_w_* and often reduce the dielectric loss factor of foods, thereby diminishing the effectiveness of emerging thermal processes, such as ohmic heating (OH) and microwave (MW) treatments. For example, Kim et al. reported that chili sauce containing 40% sugar exhibited lower inactivation efficiency under 915 MHz MW treatment than sauce with 10% sugar, as the latter showed a faster heating rate due to its higher dielectric loss factor [115]. Similarly, Park et al. demonstrated that apple juice with up to 36 °Brix (°Brix, an indicator of sugar concentration) achieved the highest inactivation efficiency owing to rapid heating during OH treatment, whereas juice with 72 °Brix showed the lowest reduction because excessive sugar suppressed electrical conductivity [116]. High sugar concentration can also enhance bacterial thermotolerance under conventional heating. Guo et al. found that supplementing tryptic soy broth with 35% sucrose increased the heat resistance of *S.* Typhimurium by upregulating the *pocR* gene involved in cobalamin biosynthesis [117]. Likewise, Alshammari et al. observed that *S.* Enteritidis PT30 exhibited higher thermal resistance in fructose, glucose and honey powder at low *a_w_* levels, with glucose conferring the greatest protection, indicating that both sugar type and matrix properties modulate bacterial tolerance [118].

Non-thermal technologies such as HPP are also impeded by high sugar concentrations, likely due to reduced *a_w_* and compressibility [119]. Scepankova et al. reported that *Bacillus subtilis* spores in undiluted honey survived after HPP treatment but were inactivated in diluted honey [120]. Similarly, Gouvea et al. observed smaller *Salmonella* reductions in açaí juice with higher sugar levels [121], while Li et al. found that D-fructose exerted a protective effect on *B. subtilis* spores during high-pressure thermal sterilization (HPTS) [122]. Other non-thermal treatments, including ultrasound (US) and pulsed light (PL), also showed decreased efficiency in high-sugar matrices [123,124], reinforcing the protective role of sugars during processing.

Conversely, only a few studies have reported sugar-enhanced microbial inactivation. Siemer et al. demonstrated that sugar-induced alterations in spore composition increased the sensitivity of *B. subtilis* spores to pulsed electric field (PEF) treatment, resulting in higher inactivation at equivalent energy levels [125].

Overall, high-sugar foods mainly pose distinctive challenges for microbial control. Their low *a_w_* and modified physicochemical characteristics often shield microorganisms from both thermal and non-thermal inactivation. Therefore, effective control strategies for high-sugar foods should account for these protective effects, employing hurdle combinations or optimized processing parameters to ensure microbial safety.

### 4.3. Impact of High-Fat Matrices on Inactivation Efficiency

**Figure 4:**
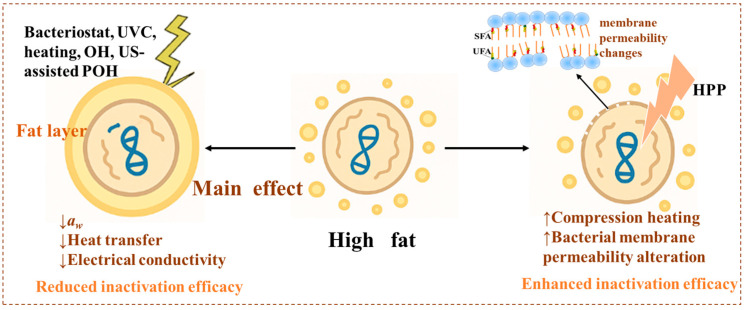
Impact of high-sugar matrices on inactivation efficiency [10,126,127,128,129,130,131,132,133,134,135,136,137,138].

The role of fat in microbial inactivation within food systems is highly complex, as it may either protect microorganisms or enhance inactivation efficiency depending on the processing method and the physicochemical nature of the food matrix (Figure 4). In most cases, high-fat environments reduce the effectiveness of both thermal and non-thermal interventions. This attenuation is primarily attributed to the physical shielding effect of fat, decreased heat transfer, lower *a_w_* during heating, and the inherently poor electrical conductivity of lipid-rich matrices. Fat can encapsulate microbial cells, forming hydrophobic barriers that hinder the diffusion of antimicrobial agents and limit the penetration of UVC radiation [8,126]. Because lipids possess lower thermal conductivity than water, they slow the rate and uniformity of heat transfer, thereby increasing the thermal resistance of pathogens such as *Salmonella* in high-fat foods [127,128,129]. In addition, the *a*_w_ of high-fat foods further decreases during heating, which in turn increases the heat resistance of microorganisms [130,131]. In pork batter, high fat content impaired the efficiency of ohmic heating due to poor electrical conductivity, resulting in lower microbial inactivation efficacy [132,133]. In milk, fat also diminished bacterial inactivation by ultrasound (US)-assisted pulsed ohmic heating (POH), reflecting its combined shielding and low-conductivity effects [134].

In contrast, the role of fat in HPP remains controversial and appears to depend on both the microorganisms and the food matrix. Rasanayagam et al. reported that higher fat levels in ground beef decreased the pressure resistance of *E. coli* strains, possibly because the compression heating of fat (approximately 8 °C/100 MPa) was higher than that of water (3 °C/100 MPa) [10,135]. Similarly, Roig-Sagués et al. found the higher fat content in milk resulted in greater lethality of *L. monocytogenes* for the same treatment conditions because milk fat alters the bacterial membrane composition and permeability [136]. Conversely, other studies indicated that fat in dry-cured ham or milk did not influence the HPP inactivation of *Salmonella* or *Bacillus* spores [137,138].

Overall, fat-rich foods often create protective microenvironments that hinder microbial inactivation. Therefore, developing effective control measures for high-fat matrices requires intensified treatments or the application of hurdle technologies to achieve reliable microbial safety.

## 5. Conclusions and Future Trends

The evidence consistently demonstrates that foodborne pathogens adopt diverse adaptive strategies to withstand the harsh stresses imposed by extreme food matrices. High-salt and high-sugar environments generally generate high osmotic pressure and low *a_w_*, prompting similar survival mechanisms such as the accumulation of osmoprotectants, modification of membrane composition, maintenance of ion homeostasis, biofilm formation, and entry into the VBNC state [139]. Under oil-rich and low-*a_w_* conditions, pathogens tend to enter a metabolically dormant state or upregulate desiccation stress-related genes to sustain long-term survival. These adaptations account for the remarkable persistence and virulence retention of pathogens in actual food matrices. Consequently, outbreaks associated with products such as chocolate, peanut butter, tahini, dried meats, and cheese remain common. Epidemiological evidence further confirms that pathogens can survive and remain infectious in these matrices for extended periods, underscoring persistent safety challenges and the urgent need for more effective inactivation strategies tailored to such extreme food systems.

In addition to affecting survival, salt, sugar, and fat profoundly influence microbial inactivation efficiency. Salt acts as a double-edged sword: while sublethal injury can increase susceptibility to subsequent hurdles such as acids or irradiation treatments, reduced *a_w_* and elevated osmotic pressure can enhance thermal tolerance and induce cross-protection. High sugar levels generally exert protective effects, decreasing the efficiency of both thermal and non-thermal treatments—including ohmic heating (OH), microwave (MW), high-pressure processing (HPP), pulsed light (PL), and ultrasound (US)—due to the reduced *a_w_*, dielectric loss factor and compressibility. Likewise, fat tends to hinder microbial inactivation by impeding heat transfer and forming protective layers. However, under HPP conditions, high fat content may instead enhance microbial lethality as a result of greater compression heating of fat- and lipid-facilitated increases in cell membrane permeability. These findings underline the importance of considering matrix-specific interactions when designing food safety interventions.

This review provides valuable insights for ensuring the microbiological safety of extreme food matrices. However, further advancements are needed to enhance the inactivation efficiency of foodborne pathogens in such challenging environments.

### 5.1. Systematic Mechanistic Studies of Microbial Survival in Real Foods

A substantial proportion of the current work studies stress adaptive mechanisms using simple systems, which poorly capture the complexity of real foods. In practice, products such as chocolate, jams, honey, syrups, and confectionery fillings simultaneously exhibit low *a_w_*, high viscosity, pH fluctuations, processing-induced changes, and interactions with multiple additives. These combined factors create heterogeneous microenvironments that can markedly alter pathogen survival and adaptation. Consequently, mechanisms inferred from simplified model systems are often difficult to extrapolate reliably to real food matrices. In addition, mechanistic studies remain fragmented. Current research often focuses on individual responses, such as compatible solute accumulation or RpoS-mediated stress regulation, without integrating the full adaptive cascade. Critical links—from osmotic stress to oxidative stress, metabolic reprogramming, entry into the VBNC or dormant state, and eventual resuscitation—are rarely examined as a connected process. Moreover, few studies combine transcriptomic, metabolomic, lipidomic and proteomic data to establish causal relationships, leaving many conclusions based on inference rather than complete mechanistic evidence. These limitations highlight the urgent need for systematic mechanistic studies in real food matrices.

### 5.2. Development of Matrix-Specific Inactivation Strategies

Salt, sugar, and fat exert profound effects on microbial inactivation behavior. Among them, salt exhibits dual effects—it can either enhance or decrease microbial inactivation depending on the environmental context and the applied inactivation treatment. These contrasting outcomes provide valuable insights for designing more targeted and efficient preservation technologies. Therefore, a comprehensive understanding of how salt influences the efficacy of different inactivation methods, which should be supported by predictive microbiology and mechanistic modeling, will be essential for achieving reliable microbial control while preserving food quality.

In contrast, high-sugar and high-fat matrices primarily pose obstacles to microbial inactivation because of their strong protective effects. Their inherent characteristics—low *a_w_*, high viscosity, and high lipid content—impair heat and mass transfer, reduce dielectric loss, and shield cells from chemical or physical stresses. Consequently, conventional treatments often fail to achieve sufficient lethality, especially in low-moisture, fat-rich products such as tahini, peanut butter, and chocolate [140]. To overcome these limitations, matrix-specific multi-hurdle strategies are required, integrating optimized combinations of technologies—for instance, high-pressure processing coupled with natural antimicrobials, ultrasound-assisted heating, or pulsed electric fields combined with mild acidification.

Future studies should focus on linking matrix composition with microbial physiology to explain differences in inactivation kinetics across food systems. Integrating these mechanistic insights into process design will enable the development of energy-efficient, matrix-tailored preservation technologies. Ultimately, a mechanism-based, model-driven framework will support the next generation of precision food preservation and help ensure the microbiological safety of high-salt, high-sugar, and high-fat foods.

## Figures and Tables

**Table 1:** Cases of bacterial contamination in high-salt foods.

Foods	Strains	Country/Year	Case/Deaths	References
Cold-smoked salmon/trout	*L. monocytogenes*	UK/2020–2024	24/4	[16]
	*L. monocytogenes*	EU (Denmark, Estonia, Finland, France, Sweden)/2014–2019	22/5	[17]
Cold-smoked fish	*L. monocytogenes*	Denmark/2013–2015	20/7	[18]
Salted egg fried rice and salted meats	*S. aureus*	Hong Kong/2019	recall	[19]
Charcuterie meat products	*Salmonella* spp.	USA/2024	104/0	[20]
Fermented, dried, and salt-cured, Italian-style meat products	*Salmonella* spp.	USA/2021	74/0	[21]
Dried pork sausages	*Salmonella.* Bovismorbificans (*S. Bovismorbificans*), *S. Typhimurium*	France/2020–2021	33/0	[22]
Antipasto trays containing assorted dry-cured meats	*S. Infanti* and *S.* Typhimurium	USA/2021	36/0	[23]
Fermented salami	*S.* Montevideo	USA/2009–2010	1656/0	[24]
Napa cabbage kimchi	*E. coli* O157:H7	Canada/2021–2022	14/0	[25]
Salt-pickled napa cabbage	*E. coli* O157:H7	Japan/2012	107/5	[26]
Fermented vegetable-Kimchi	*E. coli* O169	South Korea/2012	1642/0	[27]
Soy-sauce marinated crabs	*V. parahaemolyticus*	Korea/2018	-	[28]

## Data Availability

No new data were created or analyzed in this study.
